# Cultivation of Planktonic Anaerobic Ammonium Oxidation (Anammox) Bacteria Using Membrane Bioreactor

**DOI:** 10.1264/jsme2.ME13077

**Published:** 2013-11-08

**Authors:** Mamoru Oshiki, Takanori Awata, Tomonori Kindaichi, Hisashi Satoh, Satoshi Okabe

**Affiliations:** 1 Division of Environmental Engineering, Faculty of Engineering, Hokkaido University, North 13, West-8, Sapporo, Hokkaido 060–8628, Japan; 2 Department of Civil and Environmental Engineering, Graduate School of Engineerging, Hiroshima University, 1–4–1 Kagamiyama, Higashihiroshima 739–8527, Japan

**Keywords:** anammox, “*Candidatus* Brocadia sinica”, “*Ca*. Scalindua sp.”, enrichment, MBR

## Abstract

Enrichment cultures of anaerobic ammonium oxidation (anammox) bacteria as planktonic cell suspensions are essential for studying their ecophysiology and biochemistry, while their cultivation is still laborious. The present study aimed to cultivate two phylogenetically distinct anammox bacteria, “*Candidatus* Brocadia sinica” and “*Ca.* Scalindua sp.” in the form of planktonic cells using membrane bioreactors (MBRs). The MBRs were continuously operated for more than 250 d with nitrogen loading rates of 0.48–1.02 and 0.004–0.09 kgN m^−3^ d^−1^ for “*Ca.* Brocadia sinica” and “*Ca.* Scalindua sp.”, respectively. Planktonic anammox bacterial cells were successfully enriched (>90%) in the MBRs, which was confirmed by fluorescence *in-situ* hybridization and 16S rRNA gene sequencing analysis. The decay rate and half-saturation constant for NO_2_^−^ of “*Ca.* Brocadia sinica” were determined to be 0.0029–0.0081 d^−1^ and 0.47 mgN L^−1^, respectively, using enriched planktonic cells. The present study demonstrated that MBR enables the culture of planktonic anammox bacterial cells, which are suitable for studying their ecophysiology and biochemistry.

Anaerobic ammonium oxidation (anammox) is a microbial process in which NH_4_^+^ is anaerobically oxidized to N_2_ gas with NO_2_^−^ as an electron acceptor (20). This microbial process is mediated by anammox bacteria affiliated with a mono-phyletic group in the bacterial order *Brocadiales* in the phylum *Planctomycetes* (39). Activities and populations of anammox bacteria have been detected from various natural and engineered environments, including marine and freshwater sediments, soils, oxygen minimum zones in the ocean, wastewater treatment plants, hydrothermal vents, hot springs and petroleum reservoirs (1, 15, 54). These ubiquitous distributions of anammox bacteria are suggesting their significant contribution to the nitrogen cycle. Furthermore, anammox bacteria have uncommon physiological traits such as hydrazine synthesis (19, 50) and the presence of anammoxosome, composed of ladderane lipids (52).

Anammox bacteria have not been cultivated as a pure culture; therefore, enrichment cultures are necessary for studying their ecophysiology and biochemistry. Anammox bacteria are chemoautotrophic bacteria and their doubling time is typically 1–2 weeks (2, 11, 31, 41). Such slow growth rates have been an obstacle to obtain enrichment cultures of anammox bacteria. In addition, the batch cultivation method is not generally suitable for anammox bacteria because their growth is perturbed by their metabolites accumulating in cultures (47). Therefore, continuous cultivation methods using sequencing batch reactors (40), rotating biological contactors (11) and up-flow column reactors (7, 48) have been used for anammox bacteria. However, flotation and subsequent washout of anammox bacteria due to the production of N_2_ gas are notorious with these reactors (6, 9, 27).

Recently, membrane separation technologies have been applied to cultivate slow-growing microorganisms such as anaerobic methanotrophs (18, 26) and aerobic ammonia-oxidizing bacteria (5). Installation of membrane units having a smaller pore size than microbial cells enables full biomass retention with continuous exchange of media. For anammox bacteria, van der Star *et al.* could obtain enrichment cultures of an anammox bacterium, “*Ca.* Kuenenia stuttgartiensis”, using a membrane bioreactor (MBR) (51), in which planktonic cells dominated. Planktonic cells are suitable for physiological studies because of the homogeneity of the cell physiology. However, planktonic cells of anammox bacteria have not been obtained other than “*Ca.* Kuenenia stuttgartiensis” (51), and more attempts are required to examine the feasibility of MBR as a cultivation tool for anammox bacteria in the form of planktonic cells.

The present study was performed to obtain enrichment cultures of anammox bacteria in the form of planktonic cells using MBRs. Planktonic cells are defined in the present study as cells suspended in cultures as single cells and not settled by gravity sedimentation. For the cultivation of planktonic anammox bacterial cells, two MBRs with a hollow-fiber membrane unit were operated for >250 d after inoculation of granular sludge of “*Ca.* Brocadia sinica” and biofilms of “*Ca.* Scalindua sp.”, respectively. These biomasses were previously obtained from activated sludge at a Japanese domestic wastewater treatment plant (47) and from marine sediment in Hiroshima bay, Japan (23), respectively. The previous study revealed that “*Ca.* Scalindua sp.” is a halophilic bacterium (2), suggesting it is a member of the marine anammox species. Growth of planktonic cells of both strains was examined by fluorescence *in situ* hybridization (FISH) and 16S rRNA gene sequencing analyses. After planktonic cells were successfully obtained, the decay rate and half-saturation constant for NO_2_^−^ of “*Ca.* Brocadia sinica” were determined using enriched planktonic cells.

## Materials and Methods

### Inorganic nutrient media

Inorganic nutrient media fed into the MBR contained the following: KH_2_PO_4_ (24.4 mg L^−1^), MgSO_4_·7H_2_O (60 mg L^−1^), CaCl_2_ (51 mg L^−1^), yeast extract (Becton, Dickinson and Company, Franklin Lakes, NJ, USA) (1.0 mg L^−1^) and 0.5 ml trace element solution I and II (49). Equimolar amounts of NH_4_(SO_4_)_2_ and NaNO_2_ were supplemented into the media at final concentrations of 560–840 and 70–140 mgN L^−1^ for “*Ca.* Brocadia sinica” and “*Ca.* Scalindua sp.”, respectively. An artificial sea salt SEALIFE (Marine Tech, Tokyo, Japan) was supplemented into the media for “*Ca.* Scalindua sp.” at a final concentration of 28 g L^−1^. The detailed mineral composition of the artificial sea salt SEALIFE was described in our previous work (23). The prepared media was sparged with N_2_ gas for more than 30 min to reduce the concentration of dissolved oxygen to below 0.1 mg L^−1^ and then potassium carbonate was dissolved at a final concentration of 1.5 g L^−1^.

### MBR for “*Ca.* Brocadia sinica”

**Fig. 1:**
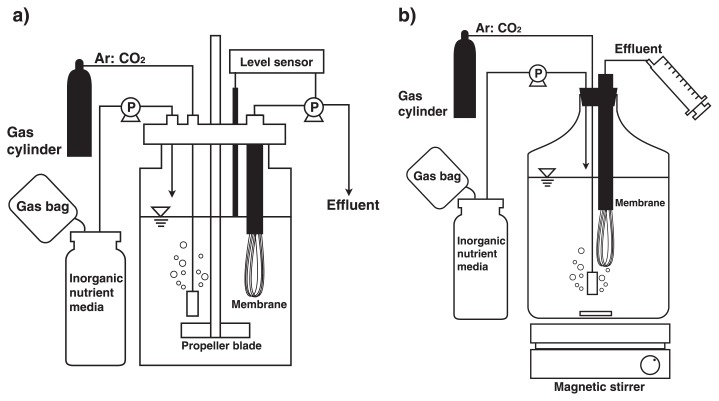
Schematic drawing of MBRs operated in the present study. a) A 3L-jar fermentor installed with a hollow-fiber membrane (0.1 μm, polyethylene) unit was used for “*Candidatus* Brocadia sinica”. Effluent was intermittently obtained by a peristaltic pump (designated as “P”) that was controlled by a level sensor. A gasbag filled with N_2_ gas was attached to the medium bottle to prevent oxygen contamination in the bottle. In addition, the reactor was continuously purged with Ar:CO_2_ (95:5) gas. b) A 2L- or 10L-glass bottle with a hollow-fiber membrane (0.4 μm, polyethylene) unit was used for “*Ca.* Scalindua sp.”. Effluent was manually collected using a disposable plastic syringe. The interior of the MBR was continuously mixed with a magnetic stirrer.

A jar fermentor MBF-500ME (volume 3L; EYELA, Tokyo, Japan) installed with a hollow-fiber membrane unit was used for cultivation of “*Ca.* Brocadia sinica” (Fig. 1a). The membrane unit was composed of 300 polyethylene tubes (pore size 0.1 μm, tube diameter 1 mm, length 70 mm) and submerged at a depth of 10 cm. Filtration was conducted using a peristaltic pump MP-1000 (EYELA) that was connected to a liquid level sensor AF-3 (EYELA). Hydraulic retention time (HRT) was set at 2 d, which resulted in a membrane flux rate of 0.011 m^3^ m^−2^ d^−1^. The culture medium in the MBR was continuously mixed with a metal propeller at 160 rpm and sparged with 95% Ar-5% CO_2_ at a flow rate of 10 mL min^−1^. Liquid temperature was maintained at 37°C with a water jacket. pH was not controlled but it ranged pH 7.6–7.8 and 7.6–8.6 for the influents and effluents, respectively. A reactor vessel was covered with thick cardboard as a light shield, which prevents the growth of phototrophic microorganisms. The membrane unit was removed every 2 weeks, washed with high-pressure tap water to detach biofilms from the membrane unit, and reinstalled in the MBR. Biomass retention time was not controlled until day 215 of operation, and thereafter a portion of culture fluid was withdrawn by the peristaltic pump to maintain the biomass retention time of 30–60 d.

As seeding biomass, granular biomass (average diameter of 2–3 mm) of *Ca.* Brocadia sinica was harvested from an up-flow column anammox reactor previously operated in our laboratory (48). This column reactor was operated with a continuous supply of inorganic nutrient media containing NH_4_^+^ and NO_2_^−^ (each 10 mM) for more than 1 year. Two hundreds milliliters (*ca.* 50 g wet) of biomass was collected using a stainless spatula and inoculated into the MBR.

### MBR for “*Ca.* Scalindua sp.”

A 2-L Pyrex screw cap storage glass bottle (Corning, NY, USA) was used as the culture vessel. This was changed to a 10-L bottle after 178 d of operation to scale up the cultures. Working volumes of cell cultures were 2 and 7 L in the 2-L and 10-L glass bottle, respectively. A hollow-fiber membrane unit was installed in the glass bottles and operated at 28°C in a dark place (Fig. 1b). The hollow-fiber membrane unit was the same as that for “*Ca.* Brocadia sinica”, except for the pore size of the membrane; *i.e.*, 0.4 μm. The culture fluid in the MBR was continuously mixed with a magnetic stirrer at 200 rpm and sparged with 95% Ar-5% CO_2_ at a flow rate of 10 mL min^−1^. pH was not controlled but it was in the range of 7.3 to 7.6. The inorganic nutrient medium was fed continuously at 1 L d^−1^, which resulted in a hydraulic retention time of 2 and 7 d in the 2-L and 10-L glass bottles, respectively. Biomass retention time was not controlled for “*Ca.* Scalindua sp.”.

A biofilm sample (*ca.* 1 g wet) taken from the previously operated up-flow column reactor equipped with a non-woven fabric sheet (23) was inoculated into the MBR after gentle homogenization.

### Microbial community analysis

FISH analysis was performed as previously described (17) to determine the abundance of anammox bacteria in enrichment cultures. Oligonucleotide probes amx820, amx368 (36) and EUB mix composed of equimolar EUB338, EUB338II and EUB338III were employed in the present study. These oligonucleotide probes were labeled with tetramethylrhodamine isothiocyanate (TRITC), Cy3, Alexa Fluor 488 or fluorescein isothiocyanate (FITC) at the 5′ end. FISH images were taken using a model LSM510 confocal laser-scanning microscope (Carl Zeiss, Oberkochen, Germany) equipped with an Ar ion laser (488 nm) and a HeNe laser (543 nm).

16S rRNA gene sequencing analysis using a Pla46f primer was performed as previously described (31). Briefly, total genomic DNA was extracted by a Fast DNA SPIN kit (MP Biomedicals, Carlsbad, CA, USA), partial nucleotide sequences ranging from the 16S rRNA to 23S rRNA gene were amplified by PCR with a primer set of Pla46f and 1037r, and the nucleotide sequences of the PCR amplicon were determined using a ABI3100-Avant Genetic Analyzer (Life Technologies, Carlsbad, CA, USA) using the Pla46f primer. Calculation of the phylogenetic tree was conducted using MEGA5.05 software (43). The partial 16S rRNA gene sequence determined by the Pla46f primer and 16S rRNA gene sequences of close relatives were aligned using Clustal W 1.83 software with the default parameters. The aligned nucleotide sequences corresponding to 220 to 827 positions on the 16S rRNA gene of *Escherichia coli* were extracted and subjected to calculation of the phylogenetic tree. Confidence of tree topology was assessed by bootstrap resampling analysis using 500 tree replicates.

### Determination of K_S_ for NO_2_^−^

The value of the half-saturation constant (*K_S_*) for NO_2_^−^ was determined as previously described (31). Briefly, planktonic cells of “*Ca.* Brocadia sinica” were collected at 215 d of operation and anaerobically incubated in 50-ml serum glass vials at 37°C with the addition of NH_4_^+^ (42 mgN L^−1^) and NO_2_^−^ (14 mgN L^−1^). Incubation was conducted for 4 h, and culture fluid samples for the determination of NO_2_^−^ were collected using a disposable plastic syringe every 15 min. Cell growth during incubation was ignored since the incubation time was much shorter than the minimum doubling times of “*Ca.* Brocadia sinica” (*i.e.*, 7 d) (31). The value of *K_s_* was calculated by the Hanes-Wool plot.

### Calculation of specific growth and decay rates

The net specific growth rate (μ*_net_*) of “*Ca.* Brocadia sinica” was calculated using planktonic cells. The value of μ*_net_* includes specific growth (μ) and decay rates (*b*) as described in Eq. 1.

(Eq. 1) $$\mu_{net}=\mu -b$$

The value of μ was calculated from the following Monod equation (Eq. 2).

(Eq. 2) $$\mu =(\mu_{max}\times S_{nitrite})/(K_{s}+S_{nitrite})$$

Where, μ*_max_*: maximum specific growth rate, *S_nitrite_*: NO_2_^−^ concentration in MBR, and *K_s_*: half-saturation constant for NO_2_^−^. For μ_max_ of “*Ca.* Brocadia sinica”, 0.0984 d^−1^ was employed in the present study, and had been previously determined from the maximum anaerobic ammonium oxidation rate and incorporation rate of ^14^CO_2_ (31). Since the inorganic nutrient media used in the present study contained NH_4_^+^ and NO_2_^−^ in an equimolar ratio, the limiting substrate was NO_2_^−^. NO_2_^−^ concentrations of effluents from the MBR during 24–217 d were used as *S_nitrite_*; *i.e.*, 0.17–0.22 mgN L^−1^. The value of *K_S_* was newly determined to be 0.47 mgN L^−1^ in the present study (see Results section). Inhibitory effects of pH, temperature and DO could be ignored because the MBR was operated at pH 7.6–8.6, 37°C and DO at <0.1 mgO L^−1^, which are favorable conditions for the growth of “*Ca.* Brocadia sinica” (31).

### Chemical analysis

Liquid samples were immediately filtrated with 0.2-μm cellulose acetate filters for the determination of NH_4_^+^, NO_2_^−^ and NO_3_^−^concentrations. Concentrations of NH_4_^+^, NO_2_^−^ and NO_3_^−^ were determined using an ion chromatograph DX-100 (Thermo Fisher Scientific, Waltham, MA, USA) with an IonPac CS3 cation column and IonPac AS9 anion column (29). In addition, NO_2_^−^ concentrations were measured colorimetrically by the naphthylethylenediamine method for cross-checking (32).

N_2_O concentration in the headspace of MBR was determined with a 1412 Photo acoustic Field Gas-Monitor (INNOVA, Copenhagen, Denmark) following the instruction manual supplied by the company. Briefly, the gas-sampling tube was connected to a sampling port of the jar-fermentor and the gas sample was continuously collected by sparging Ar gas into the MBR at a flow rate of 10 mL min^−1^.

Concentrations of total biomass and planktonic cells were determined as protein concentrations. Ten milliliters of enrichment cultures were collected from the MBR into a 10-mL glass test tube (inner diameter; 13 mm, height 105 mm) (Nichiden-Rika glass, Tokyo, Japan), and 1 ml of the well-mixed culture sample was dispensed into a 1.5 mL centrifuge tube for the determination of total biomass concentration. For planktonic cells, the rest of the enrichment culture was incubated for 5 min without shaking to settle aggregating biomass and then 1 mL supernatant was collected into another 1.5 mL centrifuge tube. The tubes were centrifuged at 14,000 rpm for 10 min, and biomass pellets were suspended in 10% (w/v) sodium dodecyl sulfate solution. After boiling for 15 min, centrifugation was performed at 14,000 rpm for 10 min and the protein concentrations of the supernatant were determined by a DC-protein assay kit (Bio-Rad, Hercules, CA, USA) according to the instruction manual.

The size distribution of aggregating biomass in enrichment cultures of “*Ca.* Scalindua sp.” was determined with a laser diffraction particle size analyzer SALD-2000J (Shimadzu, Kyoto, Japan).

### Nucleotide sequence accession number

Sequence data of the partial 16S rRNA gene for “*Ca.* Brocadia sinica” and “*Ca.* Scalindua sp.” were deposited in the DDBJ nucleotide sequence database under accession numbers AB759554 and AB822932.

## Results

### Cultivation of “*Ca.* Brocadia sinica”

**Fig. 2:**
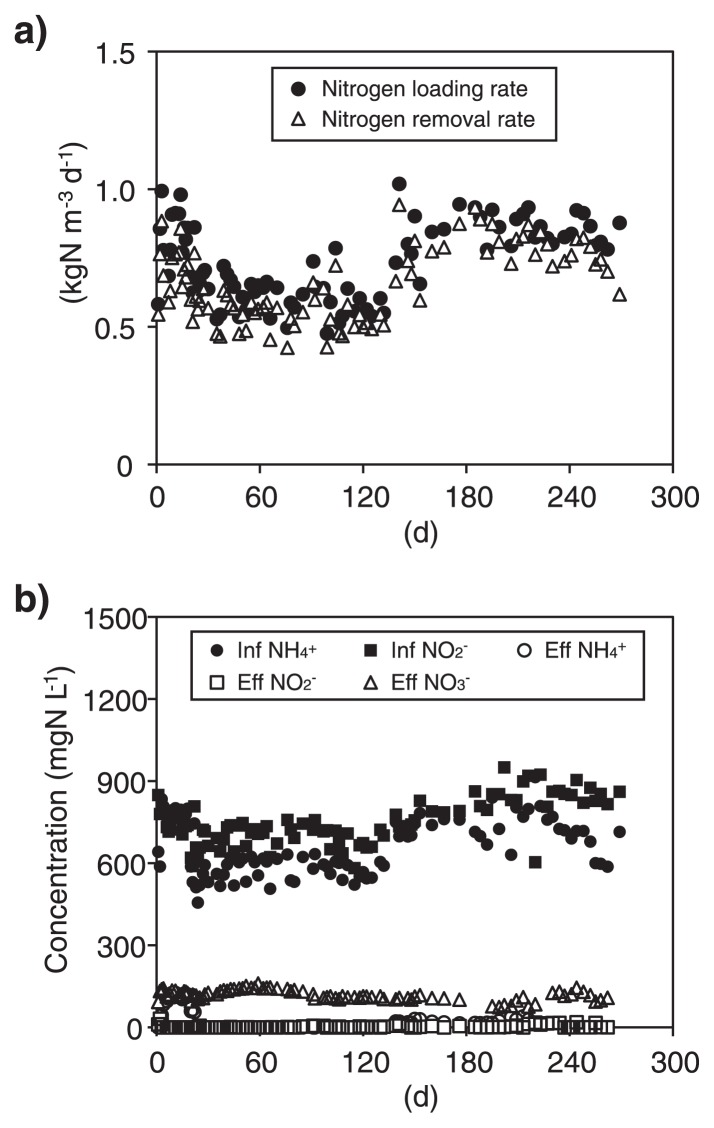
Nitrogen removal performance by enrichment cultures of “*Candidatus* Brocadia sinica”. a) Nitrogen loading (filled circle) and removal rates (empty triangle). b) Concentrations of NH_4_^+^ (circle), NO_2_^−^ (square) and NO_3_^−^ (triangle) in influents and effluents. The filled and empty symbols represent the concentrations in the influents and effluents, respectively.

An MBR was continuously operated for 269 d with nitrogen loading and nitrogen removal rates of 0.48–1.02 and 0.42 to 0.94 kgN m^−3^ d^−1^, respectively (Fig. 2a). In the MBR, both NH_4_^+^ and NO_2_^−^ were simultaneously consumed with NO_3_^−^ production (Fig. 2b), and the stoichiometric ratios of consumed NO_2_^−^ and consumed NH_4_^+^ (ΔNO_2_^−^ / ΔNH_4_^+^) and produced NO_3_^−^ and consumed NH_4_^+^ (ΔNO_3_^−^ / ΔNH_4_^+^) were 1.15–2.12 (average 1.36) and 0–0.22 (average 0.12), respectively. These values are close to the stoichiometric ratios of anammox processes (*i.e.* 1.32 and 0.26, respectively) (19, 40), indicating that the anammox process was responsible for nitrogen removal in the MBR. No serious perturbation of nitrogen removal occurred during 24–217 d of operation, and NO_2_^−^ concentrations in effluents from the MBR remained in the range of 0.17–0.22 mgN L^−1^. At 215 d of operation, N_2_O concentration in the headspace of the MBR was determined to be 44 ppm, which corresponded to 0.004% of total nitrogen concentration in influents released as N_2_O gas. This N_2_O conversion ratio was comparable to or smaller than those previously determined from anammox processes; *i.e.*, <0.01% (51), 0.03–0.06% (40) and 0.1% (30).

**Fig. 3:**
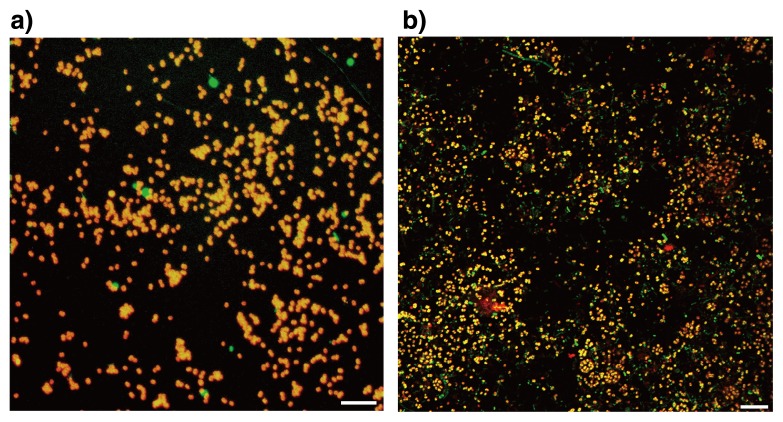
Microscopic images of fluorescence *in-situ* hybridization analysis for enriched planktonic cells of “*Candidatus* Brocadia sinica” (a) and “*Ca.* Scalindua sp.” (b). a) Enrichment cultures of “*Ca.* Brocadia sinica” were collected from the MBR after 153 d of operation and hybridized with both TRITC-labeled amx820 (red) for all the anammox bacteria and FITC-labeled EUB mix probe (green) for most members of the eubacteria. b) FISH analysis was also performed for the enrichment cultures of “*Ca.* Scalindua sp.” after 237 d of operation. Cells were hybridized with both Cy3-labeled amx368 (red) for all the anammox bacteria and Alexa Fluor 488-labeled EUB mix probe (green). Scale bar=10 μm.

**Fig. 4:**
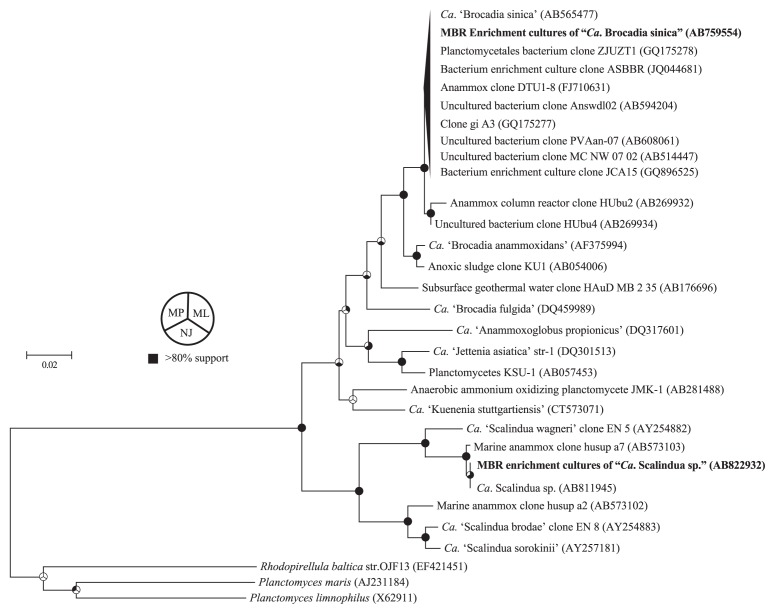
Unrooted neighbor joining tree based on the retrieved partial 16S rRNA gene sequences. Partial 16S rRNA gene sequences of “*Candidatus* Brocadia sinica” and “*Ca.* Scalindua sp.” were amplified by a primer set of Pla46f and amx1044r. The nucleotide sequences of PCR amplicon were determined using the Pla46f primer, aligned with the 16S rRNA gene sequences of the close relatives, and phylogenetic trees were calculated by the maximum likelihood (ML) method with the Kimura 2-parameter model, neighbor-joining (NJ) method with the Jukes-Cantor model and maximum parsimony (MP) method with close neighbor interchange on the random trees search algorithm. The phylogenetic tree calculated with the NJ method is shown here. The bootstrap values were calculated using 500 replicates for the ML, NJ and MP methods. Pie charts at the nodes represent the confidence of branch topology, and bootstrap values >80% are in black (MP method for upper-left sector, ML method for upper-right sector and NJ method for bottom sector).

FISH and 16S rRNA gene sequencing analysis was performed to ascertain the proliferation of “*Ca.* Brocadia sinica” in the MBR. As shown in Fig. 3a, FISH analysis with the amx820 oligonucleotide probe revealed that anammox bacteria were dominant in the MBR culture, who accounted for >90% of total biomass. Those anammox bacteria were identified as members of “*Ca.* Brocadia sinica” based on the partial 16S rRNA gene sequence determined in the present study (accession number AB759554) (Fig. 4). It should be noted that the 16S rRNA gene sequence was determined by a direct sequencing technique and no single nucleotide polymorphism was found in the sequence. This finding indicates that mono species of “*Ca.* Brocadia sinica” was enriched in the MBR.

**Fig. 5:**
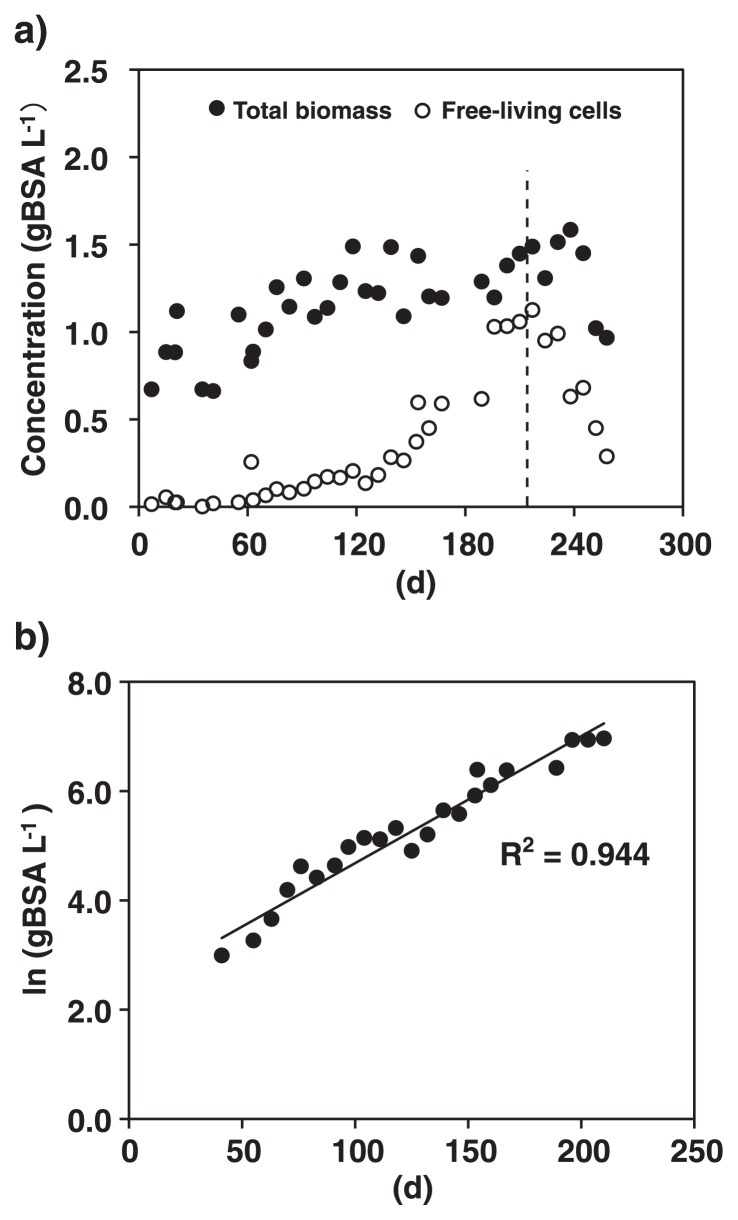
Growth trend of “*Candidatus* Brocadia sinica”. a) Concentrations of total biomass (filled circles) and planktonic cells (empty circles). A dot line at 216 d of operation indicates that control of the biomass retention time was initiated in the MBR. The biomass retention time was set at 60 and 30 d during 216–236 and 237–269 d of operation, respectively. b) Calculation of net growth rate (*μ_net_*). The concentrations of planktonic cells were plotted over time, and the value of *μ_net_* was calculated from the slope of the regression curve to be 0.0232 d^−1^.

Total biomass concentrations increased over time and reached 1.45 gBSA L^−1^ at 215 d of operation (Fig. 5a). The concentrations of planktonic cells increased from <0.1 gBSA L^−1^ at the beginning of cultivation to 1.13 g BSA L^−1^ at 215 d. As shown in Fig. 5b, the planktonic cells exponentially proliferated during 41–215 d of operation. From the slope of the regression line, *μ_net_* was determined to be 0.0232 d^−1^. The value of *μ_net_* was also estimated from the biomass retention time in the MBR. When the MBR was operated at the biomass retention time of 60 d (216–236 d of operation), the concentrations of total biomass and planktonic cells did not change; *i.e.*, 1.31–1.51 and 0.95–1.13 g BSA L^−1^, respectively (Fig. 5a). However, when the biomass retention time was decreased to 30 d after 237 d of operation, both the concentrations of total biomass and planktonic cells rapidly decreased to 0.97 and 0.29 g BSA L^−1^, respectively, at 258 d of operation. These outcomes indicated that the values of *μ_net_* were between 0.017 and 0.033 d^−1^ since the MBR was operated as chemostats where a reciprocal of the biomass retention time corresponds to the biomass growth rate required to prevent wash out of the biomass. These growth rates are comparable with the above rate calculated from the growth rate of planktonic cells; *i.e.*, 0.017–0.033 and 0.0232 d^−1^, respectively.

The *K_s_* value for NO_2_^−^ was determined to be 0.47 ± 0.3 mgN L^−1^ using enriched planktonic cells (Fig. S1). Secondly, the value of *μ* was calculated to be 0.0261–0.0314 d^−1^ by substituting *K_s_* and *μ_max_* into a Monod equation (Eq. 2). Finally, the value of *b* was calculated to be 0.0029–0.0081 d^−1^ by subtracting *μ_net_* from *μ* (Eq. 1).

Notably, planktonic cells of “*Ca.* Brocadia sinica” could be further enriched by Percoll separation, after which the cells in the culture were separated into upper and lower layers (Fig. S2), and “*Ca.* Brocadia sinica” accounted for more than 99.9% of the total biomass in the lower layer.

### Cultivation of “*Ca.* Scalindua sp.”

**Fig. 6:**
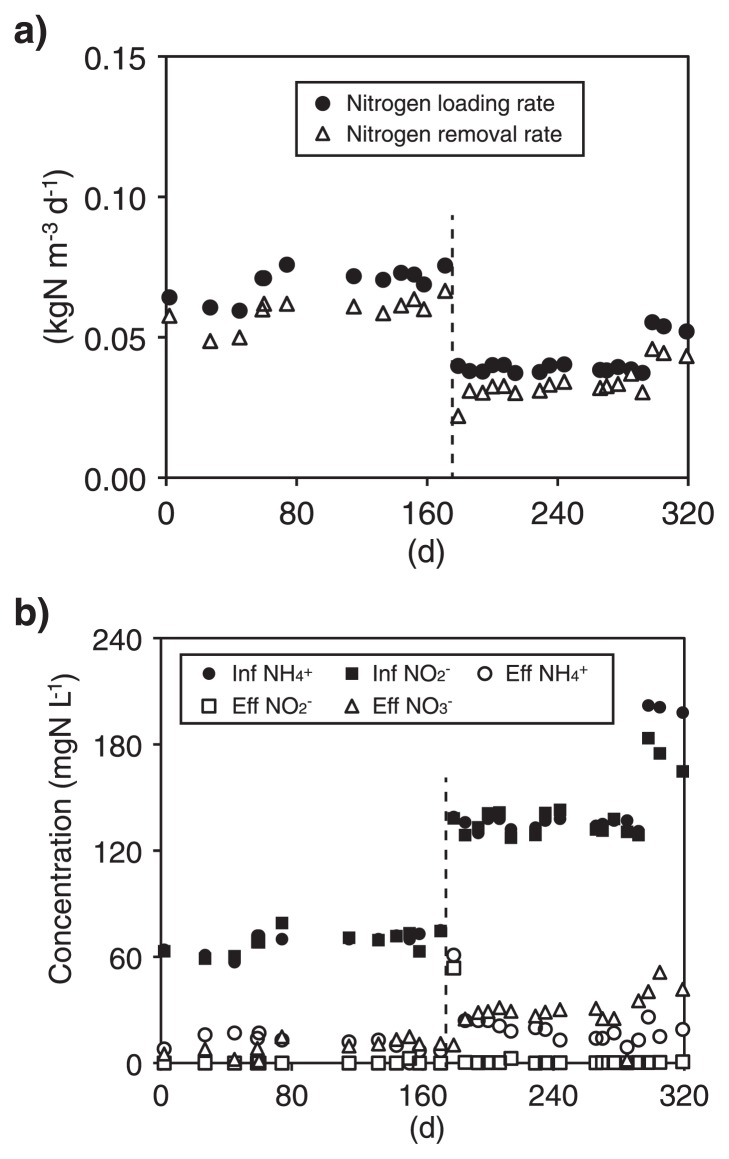
Nitrogen removal performance by enrichment cultures of “*Candidatus* Scalindua sp.”. a) Nitrogen loading (filled circle) and removal rates (empty triangle). b) Concentrations of NH_4_^+^ (circle), NO_2_^−^ (square) and NO_3_^−^ (triangle) in influents and effluents. Filled and empty symbols represent the concentrations in influent and effluent, respectively. The volume of the MBR was scaled up from 2 to 10 liters at 179 d of operation as indicated with a dotted line.

Another MBR was operated for the cultivation of “*Ca.* Scalindua sp.” with nitrogen loading rates of 0.037–0.076 kgN m^−3^ d^−1^ (Fig. 6a). At 179 d of operation, the reactor volume was scaled up from 2 to 10 liters and the nitrogen loading rate and hydraulic retention time were changed to 0.037–0.055 kg-N m^−3^ day^−1^ and 7 d, respectively. Despite this modification, stable nitrogen removal (0.022–0.067 kg-N m^−3^ day^−1^) was achieved during the entire operation. In the MBR, both NH_4_^+^ and NO_2_^−^ were simultaneously consumed with NO_3_^−^ production, and NO_2_^−^ concentrations in effluents were between 0.05–0.1 mgN L^−1^ (Fig. 6b). The stoichiometric ratios of ΔNO_2_^−^ / ΔNH_4_^+^ and ΔNO_3_^−^ / ΔNH_4_^+^ were 0.92–1.51 (average 1.13) and 0.01–0.29 (average 0.18), respectively, indicating that anammox was responsible for nitrogen removal in the MBR.

FISH analysis with the amx368 oligonucleotide probe revealed that anammox bacteria accounted for 90% of total biomass (Fig. 3b). Phylogenetic affiliation of anammox bacteria enriched in the MBR was examined by determining the 16S rRNA gene sequence with Pla46f primer. This revealed that anammox bacteria enriched in the MBR were members of “*Ca.* Scalindua sp.”, previously designated as clone husup-a7 (23) (Fig. 4).

Planktonic cells of “*Ca.* Scalindua sp.” were successfully cultivated in the MBR. Although small aggregated biomass (<100 μm) of “*Ca.* Scalindua sp.” was dominant in the cultures collected at 78 d of operation, planktonic cells dominated at 237 d of operation, as determined by microscopy (Fig. 3b). Particle size analysis revealed that particles with diameters of 0.9–1.37 μm accounted for 76% in the enrichment culture at 318 d of operation.

## Discussion

Various enrichment cultures of anammox bacteria have been previously obtained; however, they were cultivated in the form of aggregates; *i.e.*, granular sludge, flocs and biofilms (7, 22, 46). Planktonic cells are essential for studying the ecophysiology and biochemistry of anammox bacteria for the following reasons: I) the heterogeneity of the biomass aggregates with respect to physicochemical microenvironments (more active cells are present on the outside of the aggregates and less active or dormant cells are located inside) due to diffusion limitations (16, 28, 37, 38, 45), II) difficulties in extracting nucleic acids, proteins or enzymes from aggregates due to the presence of gel-like extracellular polymeric substances (EPS) (3, 8), and III) loss of activity by homogenization of the aggregate biomass. Indeed, the value of *K_s_* for NO_2_^−^ of “*Ca.* Brocadia sinica” determined in the present study was lower than that previously determined using a homogenized aggregate biomass (<100 μm) (31); *i.e.*, 0.47 ± 0.3 and 1.2 ± 0.056 mgN L^−1^, respectively. This outcome highlights the importance of planktonic cells for studying the ecophysiology of anammox bacteria. Moreover, anammox bacteria aggregates contain large quantities of EPS, as determined by microscopy (2, 22), which precluded the preparation of cell-free extract and subsequent protein purification by gel formation (8). Purified proteins are required to investigate the biochemistry of anammox pathways, and thus planktonic cells are suitable. In addition, the availability of planktonic cells enables the elimination of the procedure of biomass dispersion, which often causes a decrease of cell viability (21, 24, 34).

So far, enrichment cultures of anammox bacteria in the form of planktonic cells have been obtained from three out of five candidate genera; *i.e.*, “*Ca.* Kuenenia” (51), “*Ca.* Brocadia” and “*Ca.* Scalindua”(this study). These enrichment cultures were obtained using MBRs, indicating that MBR is an essential tool for the cultivation of planktonic anammox bacterial cells. In the present study, enrichment cultures of “*Ca.* Brocadia sinica” and “*Ca.* Scalindua sp.” were obtained after 215 and 237 d of operation, respectively. Based on *μ* of “*Ca.* Brocadia sinica” (*i.e.*, 0.0261–0.0314 d^−1^), this corresponds to 8–9 cell duplications. As for “*Ca.* Brocadia sinica”, the exponential growth of planktonic cells initiated after 60 d of operation (Fig. 5a). This lag phase is required to acclimatize their biomass to the cultivation condition employed in the MBR. The cultivation condition for “*Ca.* Scalindua sp.” was similar to that “of *Ca.* Brocadia sinica”, but differed in the following points: 1) supplementation of artificial sea salt and 2) lower nitrogen loading rate and concentrations of NO_2_^−^ and NH_4_^+^ in influents.

It should be noted that although some anammox MBRs have been previously constructed for feasibility studies on biological nitrogen removal, granular sludge or small aggregates of anammox bacteria dominated in those MBRs (42, 44, 46, 53). This finding suggests that not only complete retention of planktonic cells by membrane separation but also certain cultivation conditions are essential to obtain planktonic anammox bacterial cells. In the previous work by van der Star *et al.* (51), they pointed out that the following 4 cultivation conditions may promote the proliferation of planktonic anammox bacterial cells: I) rapid growth of anammox bacteria achieved by short biomass retention time, II) low magnesium and calcium concentrations, III) supplement of yeast extract and IV) low hydrodynamic shear stress (51). On the other hand, planktonic cells of “*Ca*. Brocadia sinica” and “*Ca*. Scalindua sp.” were obtained in the present study without controlling the biomass retention time. In addition, the value of *μ* of “*Ca.* Brocadia sinica” in the MBR was lower than their *μ_max_*; *i.e.*, 0.0261–0.0314 and 0.0984 d^−1^, respectively. These findings indicate that the rapid growth achieved by a short biomass retention time is not essential to obtain planktonic anammox bacterial cells. As for magnesium and calcium, it is well known that these divalent cation promote the precipitation of phosphorus minerals, which promote granular sludge formation because micro-organisms tend to attach and grow on their surface (46). In addition, the surface of microbial cells is typically negatively charged and divalent cations act as coagulants (33), which promotes the formation of aggregating biomass. In the present study, magnesium and calcium concentrations in the inorganic nutrient media used for the cultivation of “*Ca.* Brocadia sinica” were 6 and 18 mg L^−1^, respectively, which were comparable with those used in the previous study; *i.e.*, 9.9–39 and 41–164 mg L^−1^, respectively (51). On the other hand, these concentrations were much higher in the inorganic nutrient media used for the cultivation of “*Ca.* Scalindua sp.” due to the supplementation of artificial sea salt; *i.e.*, magnesium and calcium concentrations were 990 and 290 mg L^−1^, respectively. Therefore, the necessity of low magnesium and calcium concentrations for the cultivation of planktonic anammox bacterial cells was not as strict as previously expected. As for the influence of yeast extracts, previous studies revealed that yeast extract supplement influenced the microbial production of extracellular polymeric substances quantitatively and qualitatively (12, 13), which resulted in different cell surface properties (4). As for hydrodynamic shear stress, it is well known that high hydrodynamic shear force promotes the formation of microbial aggregates (25). In the present study, hydrodynamic shear forces were provided by mixing enrichment culture with a mechanical propeller or magnetic stirrer (160 and 200 rpm, respectively) and by sparging with Ar:CO_2_ gas (10 mL min^−1^). These conditions were comparable with those employed in the previous study (51). Another possible factor which facilitated the proliferation of “*Ca.* Brocadia sinica” in the form of planktonic cells could have been a low NO_2_^−^ concentration because aggregation is disadvantageous for microbial competition under substrate limiting conditions. The NO_2_^−^ concentrations in the MBR operated for the cultivation of “*Ca.* Brocadia sinica” were 0.17–0.22 mgN L^−1^, which were lower than the *K_s_* value for NO_2_^−^ (0.47 ± 0.3 mgN L^−1^). Specific influences of the above-mentioned cultivation conditions for the proliferation of planktonic anammox bacterial cells are still unclear, and more studies are definitely required to investigate the formation mechanisms of planktonic anammox bacterial cells in relation to cultivation conditions.

The values of *b* for “*Ca.* Brocadia sinica” were determined to be 0.0029–0.0081 d^−1^ in the present study, which were comparable with those (0.0011 and 0.0048 d^−1^) previously determined by fitting a mathematical model or batch activity test, respectively (10, 35). This indicates that the calculation of *b* using Eq. 1 and 2 and the parameters employed in those equations were reasonable. These kinetic parameters were essential to improve the system design and optimize the performance of anammox processes. For this purpose, it is important to use mathematical models (*e.g.*, an activated sludge model) (14) to understand and predict better anammox processes that cannot be observed experimentally due to the limitations of time and cost.

For further improvement of ecophysiological and biochemical studies, pure cultures of anammox bacteria are desired. It was possible to further enrich “*Ca.* Brocadia sinica” in the MBR biomass (>90%) up to 99.9% by Percoll separation (Fig. S2). This highly enriched culture is an excellent candidate for ecophysiological and biochemical studies, and even for isolation of a single cell of anammox bacteria by the serial dilution method. Future studies using a highly enriched culture will extend our knowledge of the physiological characteristics of anammox bacteria.

## Supplementary Information
