# Supplementary material for: Cultivation of Planktonic Anaerobic Ammonium Oxidation (Anammox) Bacteria Using Membrane Bioreactor
Source: Microbes Environ. 2013 Nov 8;28(4):436–43. doi: 10.1264/jsme2.ME13077 (PMC4070702; doi:10.1264/jsme2.ME13077)
Supplement: Supplementary file 1 [file 28_436_s1.pdf]

Supplemental Material

Cultivation of Planktonic Anaerobic Ammonium Oxidation (anammox) Bacteria by Using Membrane Bioreactor

By

Mamoru Oshiki[1], Takanori Awata[2], Tomonori Kindaichi[2], Hisashi Satoh[1],

and Satoshi Okabe[1,*]

[1] *Division of Environmental Engineering, Faculty of Engineering, Hokkaido University, North 13, West-8, Sapporo, Hokkaido 060-8628, Japan.*

[2] *Department of Civil and Environmental Engineering, Graduate School of Engineerging, Hiroshima University, 1-4-1 Kagamiyama, Higashihiroshima 739-8527, Japan.*

[*]*Corresponding author*

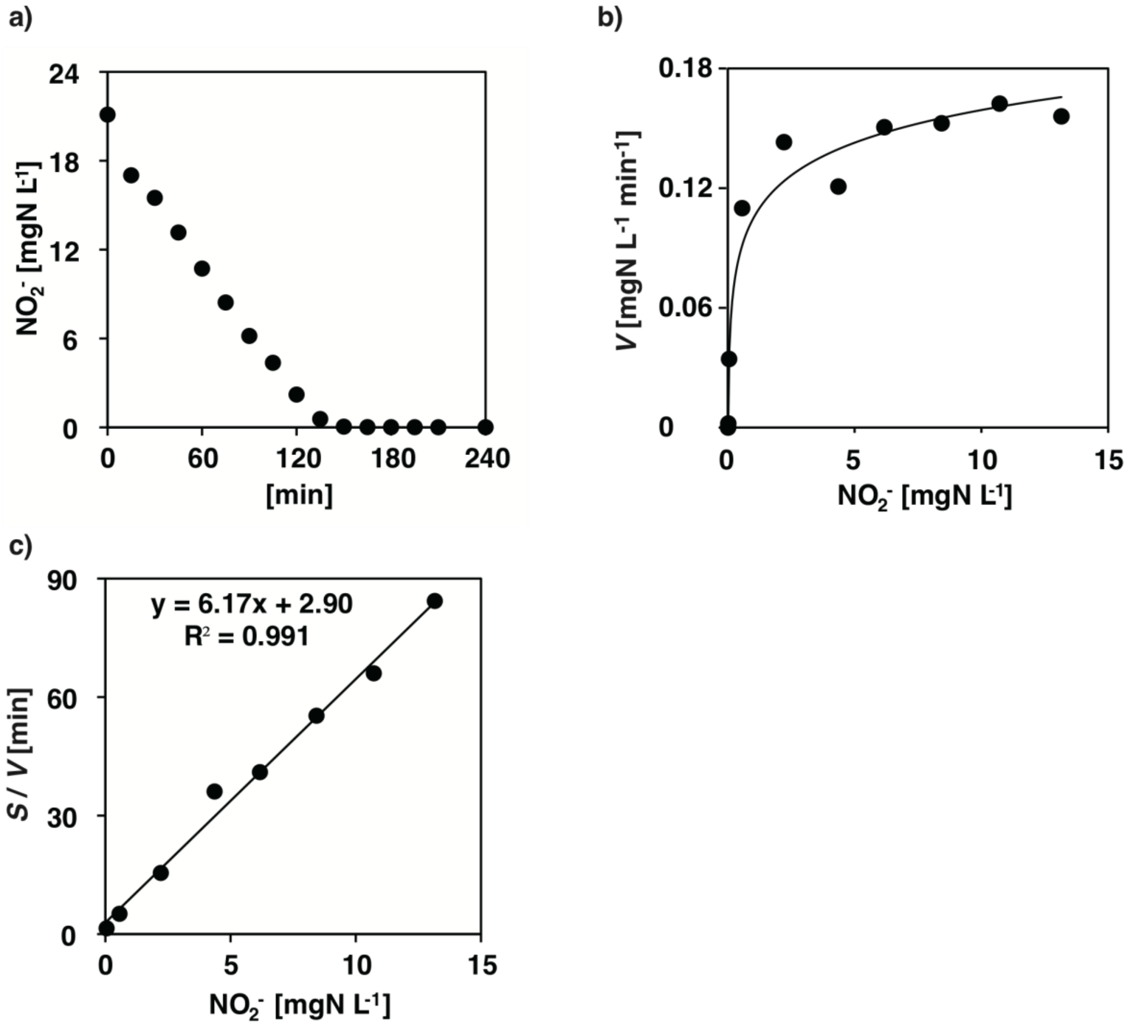

Fig. S1 Determination of K_s value for NO_2^-. Planktonic cells of "*Candidatus* Brocadia sinica" were anaerobically incubated under NO_2^- limiting condition (45 mgN L^{-1} for NH_4^+ and 21 mgN L^{-1} for NO_2^-). The concentrations of NO_2^- were determined spectrophotometrically (**a**), plotted against NO_2^- consumption rate (**b**), and the value of K_s was calculated by the Hanes-Woolf plot (**c**) where S and V indicate NO_2^- concentrations and NO_2^- consumption rate, respectively. The value of K_s was calculated as the intercept on the horizontal axis to be 0.47 ± (s.d.) 0.3 mgN L^{-1}.

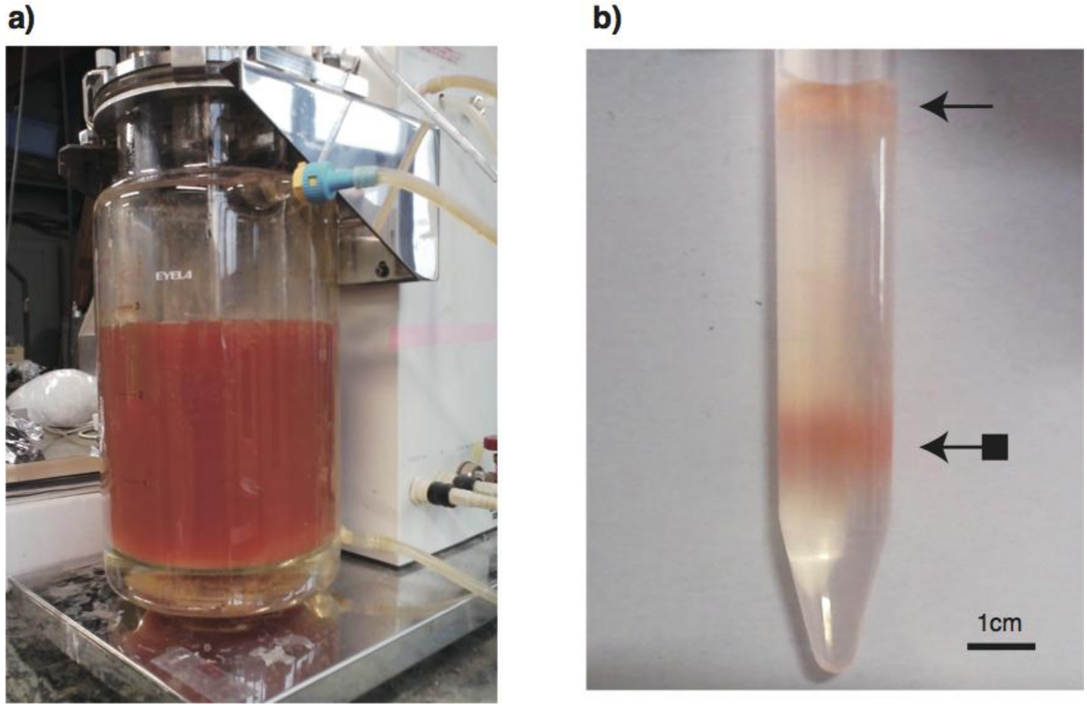

Fig. S2 Physical enrichment of "*Candidatus* Brocadia sinica". a) Highly enrichment cultures of "*Ca*. Brocadia sinica" exhibiting a vivid red color. **b)** Percoll separation of planktonic cells of "*Ca*. Brocadia sinica". The enrichment cultures were mixed with a diluted percoll solution and centrifuged at 10,000 g for 60 min (15°C). The diluted percoll solution was prepared by diluting original percoll solution (GE healthcare Japan, Tokyo, Japan) with effluent from MBR at the ratio of 2:1. The diluted percoll solution was centrifuged at 10,000g for 30 min (15°C) to prepare buoyant density gradient. After centrifugation, cells in the enrichment cultures were separated into upper and bottom layers as shown by an arrow without and with a closed square symbol at the end, respectively. "*Ca*. Brocadia sinica" accounted for more than 99.9% of total biomass in the bottom layer as determined by FISH analysis.
